# Whole-brain 3D imaging of dopaminergic neurons and glial cells in the mouse model of Parkinson’s disease induced by 6-OHDA

**DOI:** 10.3389/fnagi.2025.1503168

**Published:** 2025-03-25

**Authors:** Mengqi Wang, Linglong Xiao, Yifeng Shi, Yaping Wu, Xinyuejia Huang, Yang Wu, Yangyang Xu, Lin Bai, Wei Pan, Jie Zhang, Wei Wang

**Affiliations:** ^1^Department of Neurosurgery, West China Hospital, Sichuan University, Chengdu, Sichuan, China; ^2^Department of Neurosurgery, The First People’s Hospital of Yunnan Province, The Affiliated Hospital of Kunming University of Science and Technology, Kunming, China; ^3^Core Facility of West China Hospital, Sichuan University, Chengdu, China

**Keywords:** Parkinson’s disease, 3D pathology, whole-brain tissue-clearing, optical imaging, neural network

## Abstract

**Objective:**

Parkinson’s disease (PD) is the second most common neurodegenerative disease. Current understanding of the abnormal neural network in PD is limited, which may be one of the reasons for the lack of effective treatments. Tissue-clearing techniques allow visualization of neurons and gliocytes that form the structural basis of the abnormal neuronal network, thus enabling a deeper understanding of the pathological neuronal network in PD and contributing to the study of therapeutic strategies. The aim of this study was to create pathological maps of PD and perform 3D visualization of the neural network.

**Methods:**

We induced the PD model using 6-OHDA and a predesigned rotation test. We then performed tissue-clearing and 3D imaging of the whole-brain and brain slices of the mice using SHIELD and CUBIC.

**Results:**

The rotation test showed that the 6-OHDA group had a significant increase than the sham group. SHIELD results showed a significant reduction in tyrosine hydroxylase (TH) signals in the substantia nigra (SN) + ventral tegmental area (VTA) and caudate putamen (CPu) regions in the 6-OHDA group compared to the sham group. Additionally, we performed 3D imaging and reconstruction of astrocytes, microglia, dopaminergic neurons, and blood vessels in the SN + VTA to visualize the neuronal network.

**Conclusion:**

This study performed 3D imaging of the composition and spatial arrangement of neuronal vascular units at both macroscopic and microscopic levels, laying the foundation for the creation of a whole-brain pathological map of PD. It also provides a basis for exploring unknown neural circuits and visualizing them.

## 1 Introduction

Parkinson’s disease (PD) is the second most common degenerative disease of the central nervous system (CNS) after Alzheimer’s disease (AD). Various motor symptoms of Parkinson’s disease, such as resting tremor, hypertonia, and bradykinesia; postural balance disorders; and non-motor symptoms such as sleep disturbances, gastrointestinal dysfunction and hyposmia significantly affect patients’ quality of life and impose severe psychological and economic burden on patients and their families (Kalia and Lang, 2015). The pathogenesis of Parkinson’s disease has been investigated since the late 19th century (Kulcsarova et al., 2024). Initial anatomical studies identified key pathological features, with Brissaud (1899) first describing substantia nigra (SN) damage in 1899. Subsequent histological breakthroughs emerged when Lewy (1912) observed characteristic eosinophilic neuronal inclusions in 1912, later termed Lewy bodies by Engelhardt (2017) following their localization within the SN in 1919. The mid-20th century brought neurochemical insights, notably Ehringer’s, 1960 discovery of striatal dopamine deficiency in PD patients (Ehringer and Hornykiewicz, 1960). Modern research (Lees et al., 2009; Gibb and Lees, 1988; Hill et al., 1993; Wakabayashi et al., 1994) confirms that cardinal motor symptoms including resting tremor and hypertonia correlate with nigrostriatal dopaminergic neurons (DA) degeneration. However, these findings cannot fully account for all clinical symptoms, this could be due to limitations in our understanding of neural networks and the inability to visualize the abnormal neural networks at the onset of PD. A clearer and more comprehensive representation of the pathological changes in the neural network after the onset of PD would help deepen researchers’ understanding of the pathogenesis of PD, thereby advancing research progress in the treatment of PD.

The tissue-clearing techniques developed in recent years can reduce light scattering by tissues, make them “transparent” and improve the imaging depth of optical imaging technology in tissues, which can overcome the two-dimensional limitation of pathological imaging. The integration of tissue clearing with optical microscopy now permits the acquisition of complete, precise, and specific three-dimensional reconstructions of whole-brain neural networks. Researchers have developed diverse tissue transparency protocols to facilitate three-dimensional fluorescence imaging of biological specimens and investigations into disease mechanisms. Whole-organ imaging at this scale provides a fundamental basis for analyzing structure-function relationships in neural networks, with particular significance for neurological disease research. These advancements are proving instrumental in elucidating abnormal brain network connectivity patterns associated with PD. Kubota et al. (2017) applied CUBIC clearing to successfully localize brain metastases and systemic metastases of malignant tumors and obtain 3D topographic maps of tumor metastases that are more comprehensive than thin section localization and more detailed than imaging localization. Furthermore, tissue clearing methodologies now play a pivotal role in advancing our understanding of neurodegenerative pathologies. In a study by Ando et al. (2014), the tissue clearing technique was applied to the pathological analysis of brain tissue from patients with AD, and β-amyloid and tau protein deposited plaques, which represent typical pathological changes in AD, were comprehensively presented, and the excursions of individual neuronal axons were explained the deposited ones plaques were clearly visible. Most significantly, pioneering studies (Liu et al., 2016; Menegas et al., 2015) have implemented CLARITY-based tissue clearing in whole-brain PD mouse models, enabling ultrastructural visualization of DA synaptic terminals projecting to striatal, cortical, and amygdalar regions. This methodological innovation has further facilitated the mechanistic exploration of DA-mediated neural circuitry. These results demonstrate the great potential and promising application of transparency technology to reveal disease pathological changes at a deeper level.

Given the powerful capabilities and unique advantages of tissue clearing techniques in visualizing brain neural networks and the relatively limited application of these techniques in PD research, in this study, we combined the CUBIC and SHIELD tissue clearing techniques with a PD mouse model to construct them in 3D pathological brain maps of PD mice. In this study, we aimed to comprehensively and carefully describe the changes in the brain’s neural networks during the onset of PD, thereby laying the foundation for further elucidation of the mechanisms of PD pathogenesis and the further development of PD treatment. This study also represents a novel investigation into the application of CUBIC and SHIELD transparency techniques to treat neurodegenerative diseases.

## 2 Materials and methods

### 2.1 Experimental animals

Six- to eight-week-old male C57BL/6J mice weighing 20 g to 25 g were purchased from Beijing Huafukang Bioscience Co. All animals were maintained in a specific pathogen-free environment with constant temperature and humidity (22 ± 2°C with 55 ± 15% relative humidity; 12 h light-dark cycle). During the duration of the study, mice were fed standard chow and had free access to water.

### 2.2 Experimental PD model

In this study, 6-hydroxydopamine (6-OHDA) was used to construct the mouse model of PD. Anesthetize mice with 1.25% tribromoethanol by intraperitoneal injection (0.02 ml/g weight). The mice were placed on a stereotaxic frame (RWD, 68001, China). Prepare 6-OHDA (Sigma-Aldrich, 3 μL, 5 mg/mL) by sterile saline containing 0.02% ascorbic acid, inject into the right substantia nigra compacta (SNc) using a microliter syringe, control rate at 0.5 μL/min. Using the bregma as the coordinate origin position, the body projection of the injection site was located using the following reference coordinates: directly posterior, −3 mm; medial, +1.3 mm; and dorsal, −4.7 mm. Five minutes after completing the injection, slowly withdraw the needle.

### 2.3 Rotation test

Apomorphine (APO) was dissolved in sterile saline containing 0.02% ascorbic acid and injected intraperitoneally into mice at a dose of 0.5 mg/kg body weight. Mice were placed subcutaneously in a square chamber (40 cm^2^), and recorded the number of turns within a period (1 min). Mice with more than seven turns per minute were reported as valid PD models.

### 2.4 Stabilization to harsh conditions via intramolecular epoxide linkages to prevent degradation (SHIELD)

#### 2.4.1 PFA fixation

Mice were anesthetized with 1.25% tribromoethanol by intraperitoneal injection (0.02 ml/g body weight) and then perfused transcardially with ice-cold 1 × PBS followed by ice-cold 4% PFA solution. Dissected mouse brains were incubated in 4% PFA at 4°C with shaking overnight. Then wash the samples with 1 × PBS for at least 2 h twice at room temperature.

#### 2.4.2 Tissue clearing

This study referred to that of Park et al. (2018) described method and used the SHIELD for tissue cleaning and staining. Cleaning of the fixed tissue samples was performed using the Nuohai Tissue Clearing Kit [Cat. No.: NH210701, Nuohai Life Science (Shanghai) Co., Ltd.]. Briefly, each fixed sample was immersed in 50 mL of Clarifying Solution 1 at 37 °C for 5 days (the treatment time depends on the sample size and cleaning performance) with gentle shaking, and the Clarifying Solution 1 was refreshed every day. Each sample was then washed by immersion in 1 × PBS for 6 h with gentle shaking at room temperature, and the PBS was renewed every 2 h. Using an equal volume of Solution 2 incubate each sample for an additional 4 days at 37°C with gentle shaking, then the samples were washed in PBS as described above. Next, the cleared sample was immunostained using the SmartLable stochastic electrotransport instrument (LifeCanvas Technologies, MA, USA). SmartLabel electrophoresis was performed for 20 h for primary antibodies and 8 h for secondary antibodies. The antibodies used in this study were rat anti-GFAP (Thermo Fisher-13-0300), mouse anti-TH (BioLegend, 818001), donkey anti-rat Alexa Fluor 561-conjugated secondary antibody (Invitrogen, A48270), and donkey anti-mouse Alexa Fluor 488-conjugated secondary antibody (Jackson ImmunoResearch, 715-545-151). To match refractive index (RI), samples underwent 2-min agitation in EasyIndex (RI = 1.465; LifeCanvas Technologies, Cambridge, MA, USA) using an orbital shaker at 60 rpm. After index matching, the sample should be clear enough to be easily visible when immersed in EasyIndex. Samples were subjected to agarose gel electrophoresis (1.2% wt/vol in EasyIndex) before imaging.

#### 2.4.3 Imaging of Tiling Light Sheet Microscopy (Nuohai LS-18)

3D fluorescence imaging of the cleaned tissue was performed using the Nuohai LS-18 Tiling Light Sheet Microscope [Nuohai Life Science (Shanghai) Co., Ltd.; laser lines: 405, 488, 561, and 637 nm]. A 4 × tiled light sheet was used to illuminate the sample (Chen et al., 2020), and a 1×/0.25 NA objective (Olympus MVPLAPO) was used to collect fluorescence. Set the microscope magnification to 4×. The spatial resolution was approximately 3 μm^3^ × 3 μm^3^ × 7 μm^3^ under the chosen imaging conditions. Process the collected images using LS-18 ImageCombine software (Nuohai Life Science Co., Ltd., Shanghai) and render them using Amira (Thermo Fisher Scientific, USA).

### 2.5 CUBIC

#### 2.5.1 Clearing the brain with CUBIC

The methodology used in this study is similar to our team’s previous research (Xu et al., 2019). Corresponding improvements have been made to the CUBIC clearing process. In this study, we cleared the whole-brain and thick brain blocks. After overnight fixation with 4% PFA, wash brain samples with PBS three times, each time for 1 h, and shake at room temperature (RT). For thick brain blocks, after the washing step, cut the whole-brain into 2 mm thick blocks from the coronal side. Then it was further clarified with 1/2-Reagent-1 oscillating at 60 rpm for 3 h at 37°C under the same conditions, and then the Reagent-1 was changed every two days until satisfactory optical transparency was achieved.

Samples were gently shaken in PBS (1 h × 3) at RT and then oscillated at 60 rpm at 37°C with 1/2 Reagent-2 for 24 h. We used Reagent-2 for further clarification and replaced the solution every two days until satisfactory optical transparency was achieved. We can store the cleared samples at 4°C in reagent-2 until imaging. The same transparent clearance steps were performed for the whole-brain tissue, and the time of each step was extended appropriately according to the size of the tissue. All cleaning procedures were performed in the dark to avoid fluorescent bleaching.

#### 2.5.2 Immunofluorescence (IF) staining

Immunofluorescence staining of whole-brain and thick brain blocks was performed between Reagent-1 and Reagent-2. After clearing sample with Reagent-1, wash it with PBS for 1 h × 3 and then block it with 5% bovine serum albumin by shaking at 60 rpm at 37°C for 12 h to 1 week. Then, incubate the sample with primary antibodies (diluted in 5% BSA). In this study, three primary antibodies, namely GFAP (Abcam, ab53554, diluted 1:500), were used to label astrocytes. Iba1 (Wako, 019-19741, diluted 1:500) was used to label microglia. TH (Abcam, ab6211, diluted 1:1,000) was used to label dopaminergic neurons. The membranes were incubated with primary antibody for 24 h at 60 rpm and 37°C. For double staining, two types of non-homologous antibodies were incubated simultaneously. After incubating the primary antibodies, wash the sample with PBS for 1 h × 3, then apply the corresponding secondary antibody to the sample under the same conditions as before. After the secondary antibody incubation is complete, wash the sample again for 1 h × 3 with PBS. After the IFS was completed, the Reagent-2 clearing step was performed.

### 2.6 Statistical analysis

Data are presented as mean ± standard deviation (SD). Statistical analyses were performed using SPSS version 26.0 software and graphs were created using GraphPad Prism 9.0 software. All data were subjected to a normality test and a variance homogeneity test. If the results met a normal distribution and satisfied the test for homogeneity of variance, an unpaired *t*-test was used for statistical analysis. When the data did not follow a normal distribution, non-parametric tests were used. *P* < 0.05 was considered to indicate a significant difference.

## 3 Results

### 3.1 Successful induction of a mouse PD model using 6-OHDA

**FIGURE 1:**
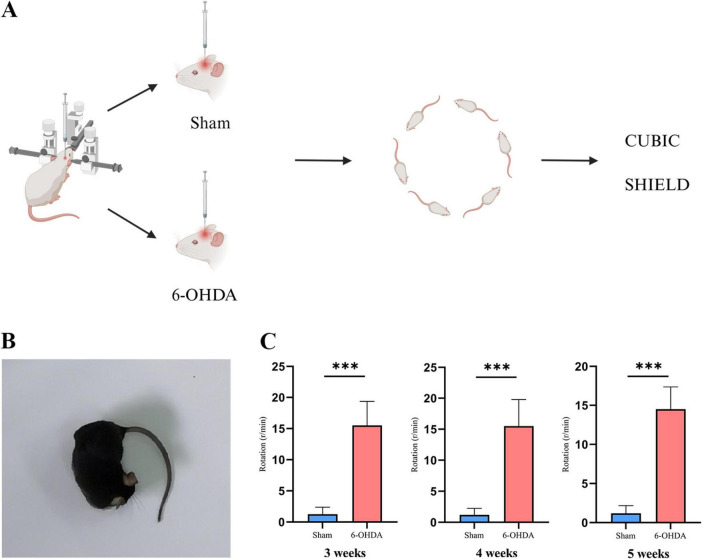
PD model construction process and behavioral assessment. **(A)** Schematic representation of the 6-OHDA-induced PD mouse model. (Created with BioRender.com). **(B)** APO-induced deflection experiment in PD mice to the opposite side of the lesion. **(C)** The results of the rotation test 3, 4, and 5 weeks after successful implementation of the PD model (***p < 0.001, *n* = 16).

To simulate clinical PD, we used 6-OHDA to induce PD in experimental mice. APO was injected intraperitoneally and the behavior of the PD model mice was assessed using a rotation test in the 3, 4, and 5 weeks after stereotactic injection of 6-OHDA. The results showed that the number of rotation circles in the 6-OHDA group was significantly greater than that in the sham group (Figure 1). There were statistical differences in the rotation test at 3 weeks (*p* = 0.000), 4 weeks (*p* = 0.000), 5 weeks (*p* = 0.001). This finding suggests that we have successfully established a PD model using 6-OHDA.

### 3.2 Multichannel labeling of mouse PD brain sections and optical imaging

**FIGURE 2:**
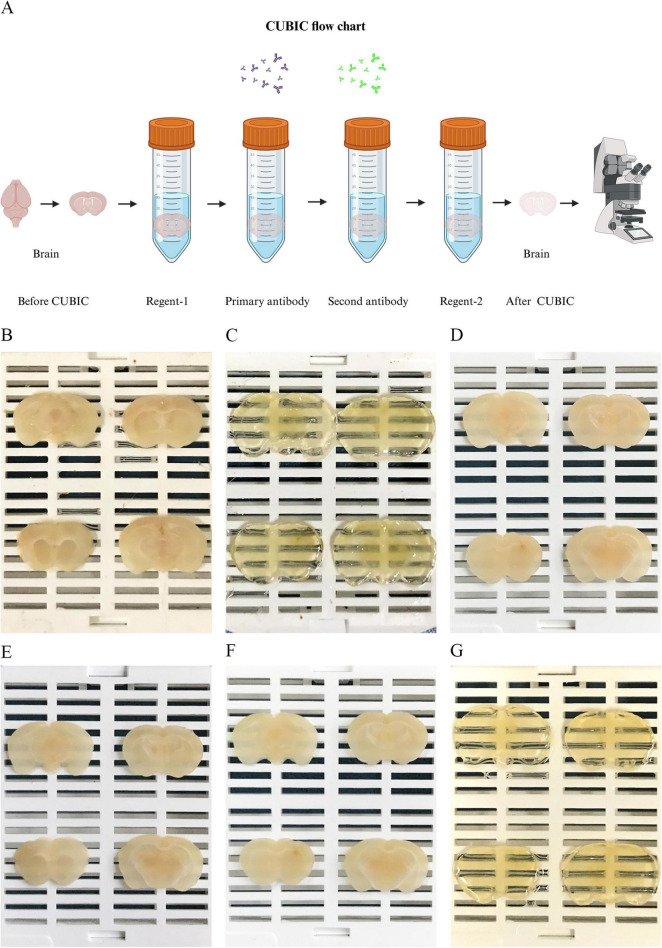
Physical representation of the CUBIC tissue clearing process. **(A)** Process and schematic diagram of CUBIC tissue cleaning. (Created with BioRender.com). **(B)** Thick brain tissue slices (1,000 μm) SN and CPU regions. **(C)** Brain tissue slices treated with Reagent-1. **(D)** Thick slices before primary antibody incubation. **(E)** Tissue slices incubated with primary antibodies. **(F)** Brain slices incubated with secondary antibodies. **(G)** Brain tissue slices treated with Reagent-2.

To investigate the pathological features of PD, we first used CUBIC combined with immunofluorescence staining to acquire data from thick coronal brain tissue sections of a PD mouse model. The process of CUBIC tissue clearing and staining is shown in Figure 2.

### 3.3 TH and GFAP staining and optical imaging of the whole-brain of PD mice from a 3D spatial perspective

**FIGURE 3:**
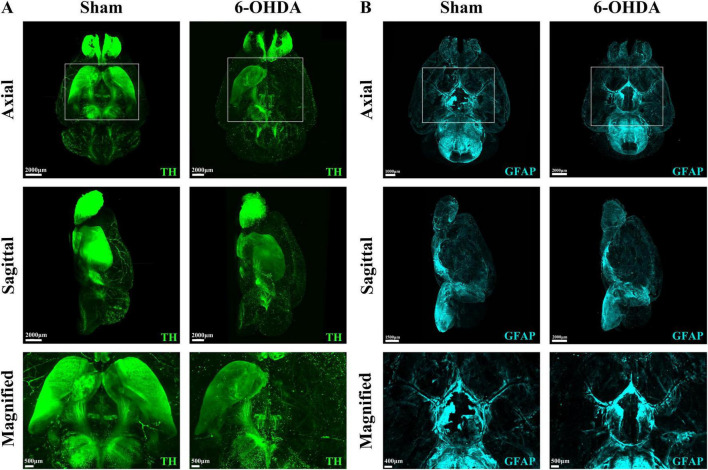
Whole-brain tissue clearing in PD mice. **(A)** 3D optical images of whole mouse brains from the Sham group and the 6-OHDA group after SHIELD tissue clearing and TH staining. **(B)** 3D optical images of whole mouse brains from the Sham group and the 6-OHDA group after SHIELD tissue clearing and GFAP fluorescent labeling.

**FIGURE 4:**
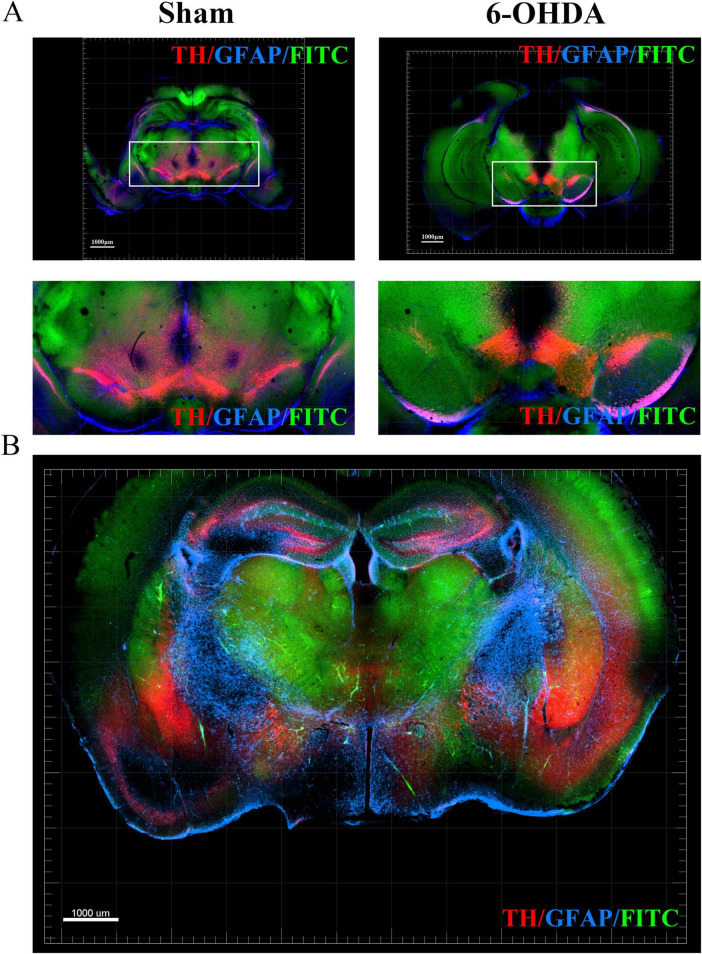
Optical imaging of the SN + VTA region in brain sections from PD mice using the CUBIC tissue clearing technique. **(A)** TH, GFAP and FITC fluorescent labeling in the SN + VTA region of the sham group and 6-OHDA group. **(B)** TH, GFAP and FITC fluorescent labeling in coronal sections.

To demonstrate the panoramic pathology of the PD brain in more detail and comprehensively, in this study, the whole-brain of PD mice was made transparent using the SHIELD tissue clearing technique combined with immunofluorescence staining for TH and GFAP labeling. The histopathological morphology of the brain of PD mice was observed from an overall macroscopic perspective (Figure 3). Compared with the Sham group, the TH fluorescence intensity in the SN + VTA area and CPu area on the ipsilateral side of injection was significantly reduced in the 6-OHDA group, whereas there was no significant change on the contralateral side (Figure 4). Furthermore, the 6-OHDA group also had significantly fewer TH-positive fibers on the same side as the sham group.

### 3.4 3D optical imaging and cell counting of dopaminergic neurons and astrocytes in the SN + VTA area of PD mice

**FIGURE 5:**
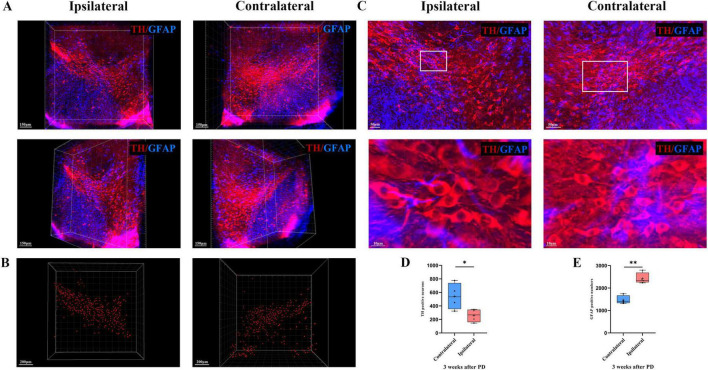
3D optical imaging of dopaminergic neurons and astrocytes in the bilateral SN + VTA region of PD mice. **(A)** Multiangle 3D optical imaging of dopaminergic neurons and astrocytes in the bilateral SN + VTA region of PD mice. **(B)** Reconstruction of dopaminergic neurons. **(C)** Cell count statistics of dopaminergic neurons and astrocytes at high magnification (per volume). **(D)** Cell count statistics of dopaminergic neurons (*p < 0.05, *n* = 4). **(E)** Cell count statistics of astrocytes (**p < 0.01, *n* = 4).

To more closely observe the morphology and number of dopaminergic neurons in the SN + VTA area of the brain of PD mice, we used the CUBIC technique to visualize the PD brain tissue and marked and observed the SN + VTA area from a microscopic perspective in a 3D view. The results showed that in the SN + VTA area of PD mice, compared with those on the contralateral side, the number of dopaminergic neurons on the ipsilateral side was significantly lower (*p* = 0.042) and the number of astrocytes was significantly higher in the 6-OHDA-injected group (*p* = 0.001) (Figure 5).

### 3.5 Optical imaging and 3D reconstruction of the spatial structural relationships of blood vessels and astrocytes in the SN of PD mice

**FIGURE 6:**
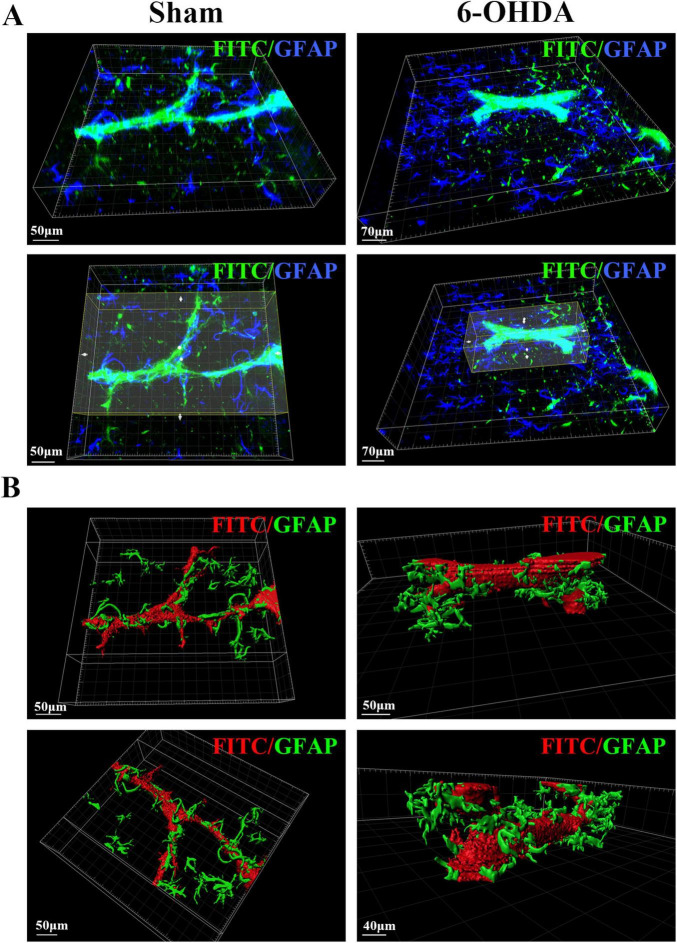
3D spatial relationship between blood vessels and astrocytes in the SN region of PD mice. **(A)** Spatial relationship between blood vessels and astrocytes in the SN region of the sham group and the 6-OHDA group. **(B)** 3D reconstruction of the spatial relationships between blood vessels and astrocytes in the SN region in the sham group and the 6-OHDA group.

To examine the changes in the neurovascular unit (NVU) in the SN area of PD mice, we labeled and optically imaged blood vessels and astrocytes in the SN area. According to the 3D spatial view, compared with those in the Sham group, the number of astrocytes around the blood vessels was larger and the cell body became thicker in the 6-OHDA group, but the contact area of the end feet with the blood vessels was reduced (Figure 6).

### 3.6 3D imaging of dopaminergic neurons, glial cells and blood vessels in the SN region of PD mice

**FIGURE 7:**
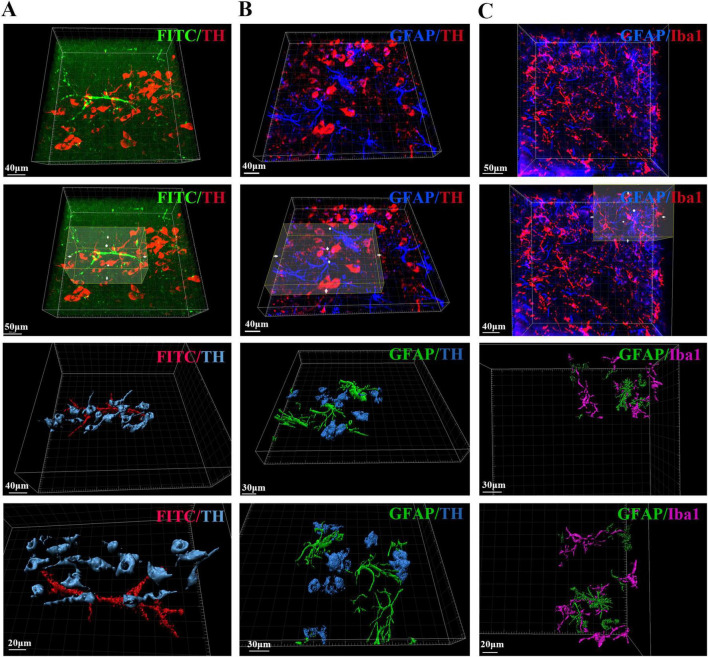
Spatial relationships between blood vessels, dopaminergic neurons, astrocytes and microglia in the SN region of PD mice. **(A)** Spatial relationship and 3D reconstruction between blood vessels and dopaminergic neurons in the SN region of PD mice. **(B)** Spatial relationship and 3D reconstruction between astrocytes and dopaminergic neurons in the SN region of PD mice. **(C)** Spatial relationship and 3D reconstruction between astrocytes and microglia in the SN region of PD mice.

In addition to blood vessels and astrocytes, the interconnectivity of glial cells, dopaminergic neurons, and blood vessels within the SN region as well as changes in their spatial positional relationships may underlie the structural basis of PD pathogenesis. To reveal their spatial relationships with each other, we also stained and labeled TH, GFAP, Iba1, and FITC and optically imaged and reconstructed them in 3D. We captured high-resolution images of dopaminergic neurons with adjacent blood vessels in the pathological state of PD after 3D rendering, as well as images of connections between astrocytes and dopaminergic neurons as well as microglia and their spatial relationships in the SN region in the pathological state of PD (Figure 7).

## 4 Discussion

Parkinson’s disease (PD) is a common neurodegenerative disease with complex pathogenic mechanisms. The central nervous system (CNS) consists of complex communication networks between cells that play a role in regulating movement, sensation, cognition and emotions (Zaman et al., 2021; Friston, 2011; Tripathi and Deogaonkar, 2020). In PD patients, changes in the structural basis of neuronal networks, including neurons, glial cells, and blood vessels, can lead to disruptions in intercellular communication and functional impairments.

Traditional pathological methods such as immunohistochemistry are widely used in PD research. Although these methods provide important morphological evidence for PD, such as dopaminergic neuron damage, they have limitations due to the thickness of the cuts. A single section cannot fully represent the changes in dopaminergic neurons in the substantia nigra and striatum in PD patients. This technique allows observation of morphological changes only from a specific perspective, without providing insights into the three-dimensional structural changes of neurons and their surrounding cells and tissues. Therefore, it is challenging to explore the underlying mechanisms and investigate changes in structure, information transmission and material transport within the neural network. With the advancement of scientific research and the continuous innovation of imaging techniques, three-dimensional imaging has become more and more suitable for studying disease mechanisms, which was previously limited to two-dimensional visualization. In recent years there have been breakthroughs in three-dimensional imaging techniques that combine high resolution with spatial structure, such as tissue clearing techniques. The emergence of tissue clearing techniques has ushered in a new era of research, allowing the observation of diverse systems, from the cellular level during embryonic development to neural circuits in the human brain (Hägerling et al., 2013; Tomer et al., 2011; Dodt et al., 2007). The complexity and heterogeneity of the nervous system have contributed to the slow progress of related research, and the emergence and development of neurodegenerative diseases affect different cell types, complicated neuronal circuits and signaling pathways. Compared to two-dimensional sections, three-dimensional imaging combined with clear tissue techniques can help reduce the difficulty and complexity of studying neurodegenerative diseases (Keller and Ahrens, 2015). In this study, we selected the relatively simple and user-friendly SHIELD and CUBIC methods from various tissue clearing techniques and applied them to PD mouse models induced by 6-OHDA. With this method, we wanted to gain a deeper understanding of the relevant pathological manifestations and structural changes from a new perspective.

We first validated the effectiveness of the model through behavioral testing, which is consistent with previous research (Lian et al., 2021). Based on the successful establishment of the PD model, we performed transparency treatment on thick brain sections by CUBIC and combined it with multichannel staining to observe the SN + VTA region from a two-dimensional perspective. We found a significant decrease in the number of dopaminergic neurons on the side injected with 6-OHDA. This approach demonstrates divergence from general two-dimensional histopathological staining techniques, such as immunofluorescence and immunohistochemistry. Traditional staining methods are limited by the inability of antibodies to penetrate deep into thick tissue, resulting in incomplete labeling of deep tissues. Therefore, traditional staining methods are more effective for thin slices. However, after making thick brain slices transparent with CUBIC and labeling them with antibodies, we can provide a more comprehensive and realistic visualization of the pathological changes in PD. In addition, based on the thick brain sections, we also performed transparency treatment on the whole mouse brain to observe the panoramic pathological morphology of PD from a three-dimensional perspective. This expands the visualization of PD research from two-dimensional to three-dimensional. Roostalu et al. (2019) performed whole mouse brain transparency in an MPTP-induced PD model, resulting in a three-dimensional visualization of the whole-brain pathology of PD. However, they only employed TH to label dopaminergic neurons and TH-positive nerve fibers and did not stain or label glial cells other than dopaminergic neurons (such as astrocytes and microglia in whole-brain), or blood vessels, particularly within key regions like the VTA and SNpc. In this study, in addition to TH, we also stained and labeled astrocytes, revealing the pathology of dopaminergic neurons and astrocytes throughout the brain in the PD state. Although our method of creating the PD model is different from theirs, it lays a visual foundation for uncovering deeper mechanisms of PD.

Furthermore, the blood vessels of the brain are a three-dimensional, anatomically and histologically heterogeneous network that transports nutrients, oxygen, metabolic wastes, signaling molecules, and drugs (Miyawaki et al., 2020). Previous studies have shown damage to the NVU and disruption of the blood-brain barrier in PD patients (Paul and Elabi, 2022). Officially introduced in 2001, the NVU concept emphasizes the close developmental, structural and functional connection between brain cells and the microvasculature and their coordinated response to injury (Iadecola, 2017). However, there is currently no research that visualizes the spatial relationship between blood vessels and neurons and glial cells in the brain in the pathological state of PD. Given the important role of damage to NVU in disease development, we labeled dopaminergic neurons, astrocytes, and blood vessels and imaged and observed brain tissue sections from a two-dimensional perspective. In addition to the macroscopic two-dimensional perspective, this study also carried out three-dimensional imaging and observation of the spatial relationships between blood vessels and astrocytes, between blood vessels and dopaminergic neurons, between astrocytes and dopaminergic neurons, and between astrocytes and microglia at the microscopic level. This is the first time that tissue clearing techniques have been applied to image and visualize blood vessels, neurons and astrocytes in the pathological state of PD in both two-dimensional and three-dimensional forms. This data set is still largely missing from the relevant literature.

In addition, this study also performed a 3D statistical analysis of TH-positive cells in the SN + VTA region of PD model mice. Our results are similar to those of Roostalu et al. (2019), although different PD model induction methods were used. However, our study achieved higher resolution and provided a clearer and more detailed representation of the dopaminergic neurons in the SN + VTA region in PD model mice.

Although tissue clearing techniques and optical imaging technologies have advanced rapidly and many scientists have developed various methods for tissue clearing, their applications in neurodegenerative diseases, including PD, are still limited. Gail Canter et al. (2019) used the SWITCH tissue clearing technique to image the spatial and temporal distribution of Aβ in the brain of 5XFAD (AD) mice, which is not possible with traditional two-dimensional tissue sections. In this study, we used the SHIELD tissue clearing technique to determine the spatial distribution of TH throughout the brain of PD model mice. Furthermore, since its development, CUBIC has been known for its imaging effectiveness and resolution from whole organ cell profiling to subcellular structures such as synapses and dendrites (Matsumoto et al., 2019). We used this method to transparentize and observe dopaminergic neurons, astrocytes, and microglia in the SN + VTA region of PD model mice by obtaining three-dimensional structural images at single-cell resolution. This creates a visual basis for depicting abnormal neural networks and neural circuits in PD patients. These results suggest that tissue clearing techniques have significant potential applications in studying the mechanisms of neurodegenerative diseases. Since abnormal neural network structures cannot be directly observed in the onset of diseases such as PD using traditional staining methods, the above transparency methods can transform abnormal neural networks from invisible to visible, providing new perspectives for revealing the underlying mechanisms of neurodegenerative diseases.

However, there are some potential limitations to this study that should be noted. First, the sample size in this study was relatively small, and future studies should increase the sample size to improve the level of evidence. Second, due to the limitations of optical technology, although clear images of the whole-brain were obtained, high-resolution images at the single-cell level were not obtained. Therefore, observations and analyses of individual cells were not performed. In addition, all of our samples were processed under the same conditions, and the spontaneous fluorescence of different samples may not be consistent. Although we have tried to minimize the differences between both as low as possible, we are still unable to completely eliminate vascular autofluorescence.

## 5 Conclusion

This study obtained neural networks in multiple brain regions through the transparency of the entire mouse brain, allowing observations and research in 2D and 3D dimensions of multiple brain regions. This study also provided detailed descriptions of the composition and spatial positioning of NVU as well as the spatial relationships between glial cells at both macroscopic and microscopic levels. This lays the foundation for the creation of a whole-brain pathological map and a whole-brain molecular pathological map of PD. In addition, this study creates a basis for research into unknown neural circuits and their visualization.

## Data Availability

The raw data supporting the conclusions of this article will be made available by the authors, without undue reservation.
